# Análise espaço-temporal da leishmaniose visceral canina em Uruguaiana, Rio Grande do Sul, Brasil (2009-2019)

**DOI:** 10.1590/0102-311XPT153625

**Published:** 2026-10-05

**Authors:** Júlia Birnie Farias Covatti, Laura Ilarraz Massia, José Ricardo Wellicks, Rita Daniela Quevedo Lamadril, Debora da Cruz Payão Pellegrini

**Affiliations:** 1 Escola Nacional da Saúde Pública Sergio Arouca, Fundação Oswaldo Cruz, Fiocruz, Rio de Janeiro, Brasil.; 2 Secretaria Municipal de Saúde, Prefeitura Municipal de Uruguaiana, Uruguaiana, Brasil.; 3 Universidade Federal do Pampa, Uruguaiana, Brasil.

**Keywords:** Leishmaniose Visceral, Sistema de Informação Geográfica, Saúde Única, Visceral Leishmaniasis, Geographic Information Systems, One Health, Leishmaniasis Visceral, Sistemas de Información Geográfica, Salud Única

## Abstract

O objetivo do estudo foi analisar a distribuição espaço-temporal dos casos confirmados de leishmaniose visceral canina (LVC) no Município de Uruguaiana, Rio Grande do Sul, Brasil, no período de 2009 a 2019. Utilizou-se a estatística de varredura para a detecção de aglomerados espaço-temporais de casos, por meio do programa SaTScan. O banco de dados foi composto por registros provenientes de investigações por demanda espontânea e inquéritos sorológicos, totalizando 3.339 cães investigados, dos quais 1.478 foram classificados como sororreagentes para LVC. Foram identificados cinco aglomerados espaço-temporais ao longo do período analisado, concentrados em setores censitários específicos do município, com risco relativo elevado em comparação às demais áreas. Os achados evidenciam a heterogeneidade da transmissão no território urbano e reforçam a utilidade da análise espaço-temporal para subsidiar ações territorializadas de vigilância e controle.

## Abstract (en)

This study aimed to analyze the spatiotemporal distribution of confirmed cases of canine visceral leishmaniasis in Uruguaiana, Rio Grande do Sul State, Brazil, from 2009 to 2019. Scan statistics were used on SaTScan program to detect spatiotemporal case clusters. The database was composed of records from spontaneous demand investigations and serological surveys, totaling 3,339 investigated dogs, of which 1,478 were classified as seroreagents for canine visceral leishmaniasis. This study found five spatiotemporal clusters over the analyzed period in the census tracts of the municipality with high relative risk than other areas. The findings show the heterogeneity of transmission in the urban territory and reinforce the usefulness of spatiotemporal analysis to support territorialized surveillance and control actions.

## Abstract (es)

Este estudio tuvo como objetivo analizar la distribución espaciotemporal de los casos confirmados de leishmaniasis visceral canina (LVC) en el municipio de Uruguaiana, Rio Grande do Sul, Brasil, entre 2009 y 2019. Se utilizó la estadística de barrido para detectar conglomerados espaciotemporales de casos, mediante el programa SaTScan. La base de datos estuvo compuesta por registros provenientes de investigaciones por demanda espontánea y encuestas serológicas, con un total de 3.339 perros investigados, de los cuales 1.478 se clasificaron como seropositivos para LVC. Se identificaron cinco conglomerados espaciotemporales a lo largo del período analizado, concentrados en sectores censales específicos del municipio, con un riesgo relativo alto en comparación con las demás áreas. Los hallazgos demuestran la heterogeneidad de la transmisión en el territorio urbano y refuerzan la utilidad del análisis espaciotemporal para respaldar acciones territorializadas de vigilancia y control.

## Introdução

A leishmaniose figura entre as dez principais doenças tropicais negligenciadas e mantém circulação em quatro grandes regiões ecoepidemiológicas - Américas, Leste da África, Norte da África e Ásia Ocidental/Sul-Sudeste - com 80 países classificados como endêmicos em 2024 ^1^. Trata-se de uma condição potencialmente fatal, podendo evoluir para óbito em mais de 90% dos casos quando não tratada, e cujo tratamento, ainda que recomendado pelas diretrizes vigentes, apresenta desafios importantes relacionados à eficácia, à toxicidade e ao acesso. No Brasil, a leishmaniose visceral (LV) acomete com maior frequência crianças com até dez anos de idade; contudo, tem-se observado crescente relevância clínica associada à coinfecção HIV/LV, padrão semelhante ao descrito em alguns países europeus ^2^.

As epidemias urbanas de LV foram observadas em diversas cidades brasileiras e a rápida expansão geográfica está associada com vários determinantes, tais como mudanças ambientais, intensas migrações responsáveis por ocupações desordenadas e condições precárias de vida nos centros urbanos ^3^ ^,^ ^4^ ^,^ ^5^. O contato estreito entre seres humanos e animais reforça a necessidade de adotar a abordagem Saúde Única (*One Health*) para estratégias de controle e mitigação mais efetivas, considerando-se a interdependência entre as saúdes humana, animal e dos ecossistemas coabitados.

Pelo potencial zoonótico, a leishmaniose visceral canina (LVC) se destaca como uma enfermidade parasitária relevante no Brasil ^6^ ^,^ ^7^ ^,^ ^8^ ^,^ ^9^ devido às características clínicas e de transmissibilidade ^10^ ^,^ ^11^ ^,^ ^12^, pois a enzootia canina tem precedido a ocorrência de casos humanos, apresentando maiores índices de prevalência ^8^ ^,^ ^13^.

Atualmente, as estratégias de controle vigentes para LV estão fundamentadas no diagnóstico e tratamento precoce dos casos humanos, na redução da população de flebotomíneos, na eutanásia de casos caninos confirmados, além de atividades de educação em saúde ^14^. Entretanto, a aplicação intensiva dessas medidas não tem sido suficiente para interromper a transmissão em áreas urbanas, indicando a necessidade de novas estratégias de vigilância que permitam delimitar áreas de maior risco e avaliar a efetividade das ações de controle no território.

Até novembro de 2008, o Rio Grande do Sul era considerado área indene para LV, quando foi identificado um cão sororreagente em São Borja, seguido da confirmação do primeiro caso humano no estado. A partir da comprovação da autoctonia, diversos municípios da fronteira oeste iniciaram investigações epidemiológicas, registrando a presença do vetor *Lutzomyia longipalpis* ^14^, cães sororreagentes e a caracterização de *Leishmania infantum* em amostras caninas.

No Município de Uruguaiana, o primeiro registro canino ocorreu em 2009, desencadeando ações de vigilância baseadas principalmente no diagnóstico de cães. Apesar de o município ter registrado quatro casos humanos desde 2011 e apresentar intensa circulação transfronteiriça − sendo considerado área estratégica para a vigilância no estado −, a distribuição espaço-temporal da LVC no território municipal permanece pouco conhecida, constituindo uma importante lacuna para o planejamento das ações de controle.

O mapeamento de doenças constitui uma das aplicações mais comuns dos sistemas de informações geográficas (SIG) na epidemiologia, por permitir a representação e análise da distribuição espacial dos agravos, facilitando a identificação de padrões de aglomeração e áreas de risco. Estudos prévios apontam dependência espacial da LVC e da LV no Brasil e em outros países ^15^ ^,^ ^16^.

Diante desse cenário, o presente estudo tem como objetivo descrever a soropositividade para LVC e analisar a distribuição espaço-temporal de cães sororreagentes no Município de Uruguaiana, entre 2009 e 2019, por meio da estatística de varredura espaço-temporal (SaTScan; http://www.satscan.org).

## Desenvolvimento

### Desenho de estudo e fonte dos dados

Foi realizado um estudo ecológico, com base em dados secundários referentes aos casos notificados de LVC no período de janeiro de 2009 a dezembro de 2019, cedidos pelo Setor de Vigilância Ambiental em Saúde (VAS) da Secretaria Municipal de Saúde de Uruguaiana.

Os registros incluíram informações provenientes tanto da vigilância passiva (demanda espontânea), caracterizada pela procura voluntária dos tutores para investigação diagnóstica, quanto da vigilância ativa (inquéritos sorológicos), realizadas em decorrência da detecção de casos caninos ou humanos de LV. As diferenças metodológicas e de cobertura territorial entre essas modalidades de vigilância são apresentadas no Material Suplementar (https://cadernos.ensp.fiocruz.br/ojs/index.php/csp/article/view/10769/21099).

Os inquéritos caninos foram conduzidos conforme as diretrizes do *Manual de Vigilância e Controle da Leishmaniose Visceral* do Ministério da Saúde ^14^, envolvendo a busca ativa de cães domiciliados em áreas previamente delimitadas e a realização de exames sorológicos segundo o protocolo vigente em cada período.

Foram considerados sororreagentes para LVC os cães positivos no protocolo diagnóstico adotado no respectivo ano. Entre 2009 e 2013, utilizou-se o teste rápido DPP (domicílios particulares permanentes) como triagem e RIFI (reação de imunofluorescência indireta) como confirmatório, a partir de 2014, adotou-se DPP seguido de ELISA (*enzyme-linked immunosorbent assay*) como teste confirmatório.

Foram incluídos no estudo casos caninos com confirmação laboratorial para LVC, residentes na zona urbana do município e com endereço completo, permitindo a sua alocação aos respectivos setores censitários. Foram excluídos os casos com endereço incompleto, localizados na zona rural ou não confirmados laboratorialmente.

As bases cartográficas dos setores censitários urbanos foram obtidas no *Censo Demográfico* de 2010 ^17^ do Instituto Brasileiro de Geografia e Estatística (IBGE).

### Área de estudo

O Município de Uruguaiana está situado na Região da Campanha Ocidental, no extremo oeste do Rio Grande do Sul, Brasil. Segundo o *Censo Demográfico* de 2010 ^17^, tem 117.210 habitantes distribuídos entre zona urbana e rural, com clima subtropical caracterizado por invernos frios e verões de elevadas temperaturas.

Trata-se de área estratégica sob o ponto de vista geopolítico e econômico devido à intensa circulação transfronteiriça e à relevância na produção agropecuária nacional, especialmente na orizicultura ^18^.

A zona urbana do município é composta por 118 setores censitários, que constituíram a unidade espacial de análise adotada neste estudo.

### Georreferenciamento dos casos

As coordenadas geográficas (latitude e longitude) dos casos confirmados de LVC foram obtidas individualmente no programa Google Earth Pro (versão 7.3.3.7786; https://earth.google.com), por meio da ferramenta de marcação de pontos, e posteriormente agregadas aos respectivos setores censitários urbanos.

Reconhecem-se as limitações inerentes ao georreferenciamento manual, incluindo possíveis imprecisões na definição das coordenadas e pequeno deslocamento espacial residual. Optou-se pela utilização dessa ferramenta devido à indisponibilidade de registros geográficos oficiais padronizados, assumindo-se os potenciais impactos dessas imprecisões na interpretação dos padrões espaciais identificados.

### Análise espacial descritiva

Para a caracterização da distribuição territorial dos casos, foram elaborados mapas coropléticos representando a distribuição dos cães sororreagentes segundo setor censitário urbano, discriminando-se os dados provenientes da vigilância passiva e da vigilância ativa.

A unidade espacial de análise foi o setor censitário urbano. Foram representados os números absolutos de casos por setor ao longo do período estudado (2009-2019).

Os mapas foram construídos no software QGIS (versão 3.10.7; https://qgis.org/en/site/).

### Análise espaço-temporal inferencial

A análise espaço-temporal foi realizada utilizando-se o software SaTScan (versão 10.0.2, 64 bit; http://www.satscan.org), por meio da estatística de varredura espaço-temporal proposta por Kulldorff ^19^, sob modelo discreto de Poisson. Foram incluídos exclusivamente os casos confirmados provenientes da vigilância passiva (demanda espontânea) que apresentavam endereço completo no perímetro urbano do município, com o objetivo de assegurar comparabilidade temporal e uniformidade territorial ao longo da série histórica, uma vez que os inquéritos sorológicos ocorreram em anos específicos e com delimitação geográfica distinta.

No modelo, foram utilizados como numerador os casos confirmados de LVC agregados por setor censitário e ano de ocorrência, e como denominador o número de domicílios particulares permanentes por setor censitário, obtido no *Censo Demográfico* de 2010, usado como *proxy* da população sob risco.

Foram definidos como limites máximos 25% da população sob risco e 50% do período total do estudo (2009-2019), considerando-se o ano calendário como unidade temporal.

Optou-se por calcular o risco relativo (RR) como medida de associação, bem como os respectivos valores de p (p < 0,05) para a identificação de aglomerados espaço-temporais estatisticamente significativos.

### Análises espaciais exploratórias

Como análises espaciais exploratórias complementares, foram empregadas a estimativa de densidade Kernel e os índices Global e Local de Moran, com o objetivo de avaliar os padrões de agregação espacial dos casos provenientes da vigilância passiva ao longo dos anos.

A estimativa de Kernel foi utilizada para gerar superfícies contínuas de densidade e identificação visual de áreas de maior concentração de casos. Os índices Global e Local de Moran foram aplicados para verificar a existência de autocorrelação espacial global e identificar agrupamentos do tipo “Alto-Alto”.

Essas análises têm caráter exploratório e auxiliar, sendo apresentadas no Material Suplementar (https://cadernos.ensp.fiocruz.br/ojs/index.php/csp/article/view/10769/21099).

### Aspectos éticos

O estudo foi aprovado pelo Comitê de Ética em Pesquisa da Universidade Federal do Pampa (sob o registro nº 58534316.9.0000.5323).

## Resultados

No período de 2009 a 2019, foram realizadas 3.339 investigações para LVC no Município de Uruguaiana. Desse total, 68,07% (n = 2.273) corresponderam à vigilância passiva e 31,93% (n = 1.066) à vigilância ativa, realizada por meio de inquéritos sorológicos.

### Vigilância ativa (inquéritos sorológicos)

Entre 2009 e 2010, foram conduzidos sete inquéritos sorológicos após a identificação dos primeiros casos caninos no município, abrangendo os bairros Nova Esperança, Tarragó, São Miguel, Tabajara Brites, São João, Mascarenhas de Morais, Santo Inácio e COHAB II. Posteriormente, novos inquéritos foram realizados em 2011, 2016 e 2017 em decorrência da ocorrência de casos humanos de LV nos bairros Mascarenhas de Morais, São Miguel e São João.

A proporção média de cães sororreagentes nos inquéritos realizados ao longo do período foi de 6,57%. O maior número de coletas ocorreu em 2009 (584 amostras), com 1,2% de positividade, e a maior proporção de soropositividade foi registrada em 2016 (48%). Observou-se maior positividade nos inquéritos vinculados à ocorrência de casos humanos (2011, 2016 e 2017). A distribuição anual das investigações e da sororreatividade é apresentada na Tabela 1.

**Tabela 1:** Distribuição anual das investigações para leishmaniose visceral canina (LVC), segundo tipo de vigilância (ativa e passiva), número de cães examinados, número de sororreagentes e respectivas proporções. Município de Uruguaiana, Rio Grande do Sul, Brasil, 2009-2019.

Ano	Demanda espontânea			Inquéritos		
	Amostras	Sororreagentes	%	Amostras	Sororreagentes	%
2009	0	0	0,0	584	7	1,2
2010	126	28	22,2	378	29	7,7
2011	281	105	37,4	28	7	25,0
2012	392	262	66,8	-	-	-
2013	500	373	74,6	-	-	-
2014	248	195	78,6	-	-	-
2015	228	192	84,2	-	-	-
2016	184	129	70,1	25	12	48,0
2017	119	77	64,7	51	15	29,4
2018	69	40	58,0	-	-	-
2019	126	77	61,1	-	-	-

### Vigilância passiva (demanda espontânea)

Foram analisadas 2.273 investigações provenientes de demanda espontânea, das quais 1.478 (65,02%) apresentaram sororreatividade para LVC. No ano de 2009, não houve coletas por demanda espontânea, estando as investigações restritas aos inquéritos sorológicos.

O maior número de amostras por demanda espontânea foi registrado em 2013 (500 coletas, com 74,6% de cães sororreagentes), já o menor número ocorreu em 2018 (69 coletas, com 57,97% de positividade). A maior proporção de soropositividade foi observada em 2015 (84,21%). A partir de 2012, a frequência anual de soropositividade manteve-se superior a 58%.

### Distribuição espacial por setor

A distribuição espacial acumulada dos casos confirmados provenientes da vigilância passiva (2009-2019), segundo setor censitário urbano, evidenciou ampla disseminação territorial da LVC no município. Observou-se maior concentração de casos em setores localizados principalmente nos bairros Nova Esperança, São João e Cabo Luís Quevedo, que apresentaram valores mais elevados no período acumulado. No que se refere à vigilância ativa, os inquéritos apresentaram cobertura territorial mais delimitada, concentrando-se nos setores onde foram identificados casos caninos ou humanos, refletindo o caráter focal dessa estratégia de vigilância (Figura 1).

**Figura 1:**
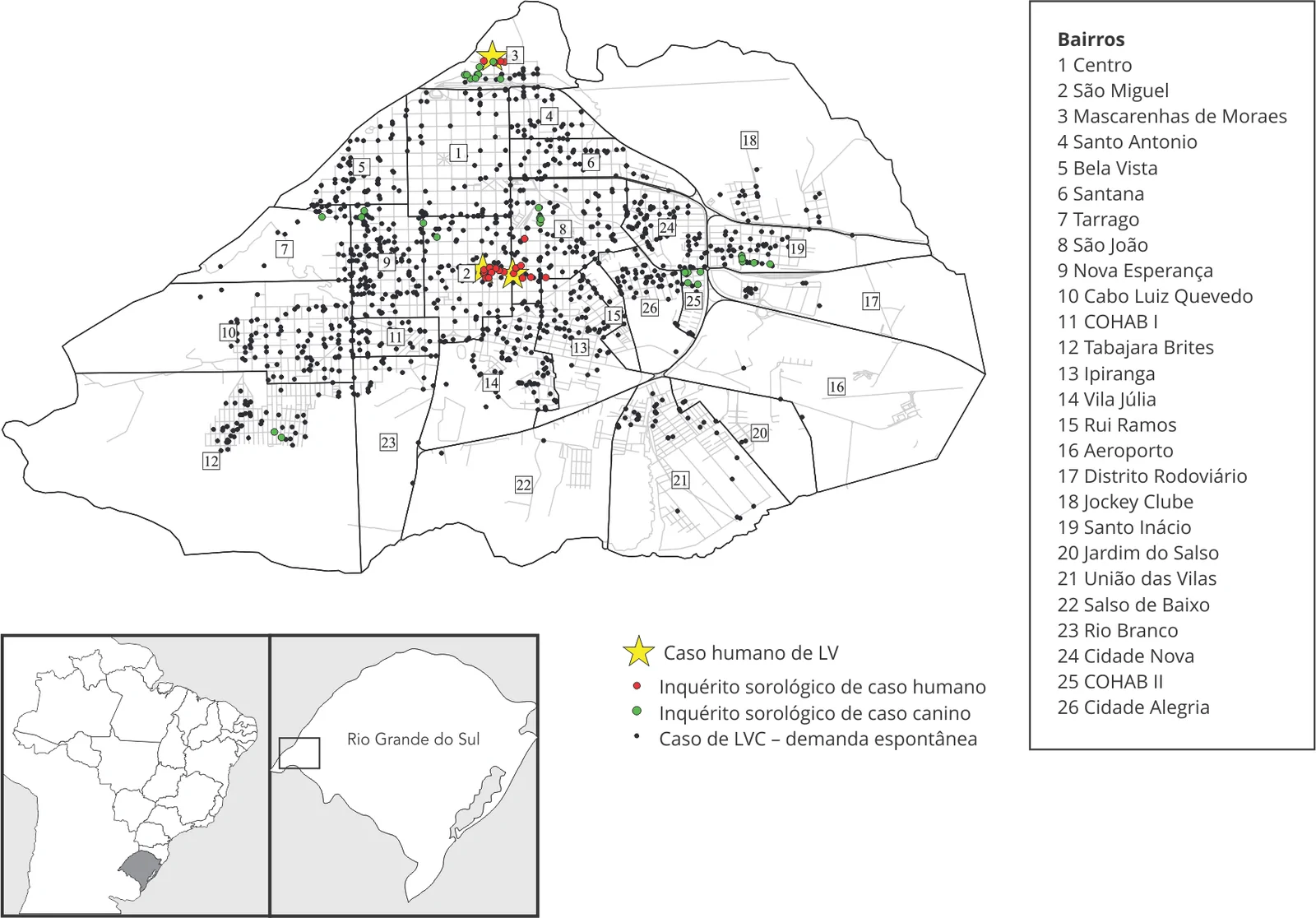
Distribuição espacial dos casos de leishmaniose visceral canina (LVC) identificados por vigilância passiva e por inquéritos sorológicos, bem como dos casos humanos de leishmaniose visceral (LV). Município de Uruguaiana, Rio Grande do Sul, Brasil, 2009-2019.

### Análise espaço-temporal

Considerando a ampla distribuição territorial observada na análise descritiva, procedeu-se à análise espaço-temporal por meio da estatística de varredura.

Para essa análise foram considerados exclusivamente os casos confirmados provenientes da vigilância passiva com endereço completo (n = 1.394). A razão acumulada de casos de LVC no período analisado (2010-2019), em relação ao total de domicílios particulares permanentes, foi de 4,03 casos por 100 domicílios.

Foram identificados cinco aglomerados espaço-temporais estatisticamente significativos no período de 2009-2019 (Figura 2; Tabela 2). Esses aglomerados envolveram setores censitários contíguos, configurando áreas de maior concentração de casos no território urbano.

O primeiro caso humano de LV ocorreu no bairro Mascarenhas de Morais, em 2011, coincidindo temporal e espacialmente com o aglomerado 5. Da mesma forma, os casos humanos registrados em 2016 e 2017 coincidiram com o aglomerado 3, sugerindo sobreposição entre áreas de maior risco canino e ocorrência de casos humanos.

**Figura 2:**
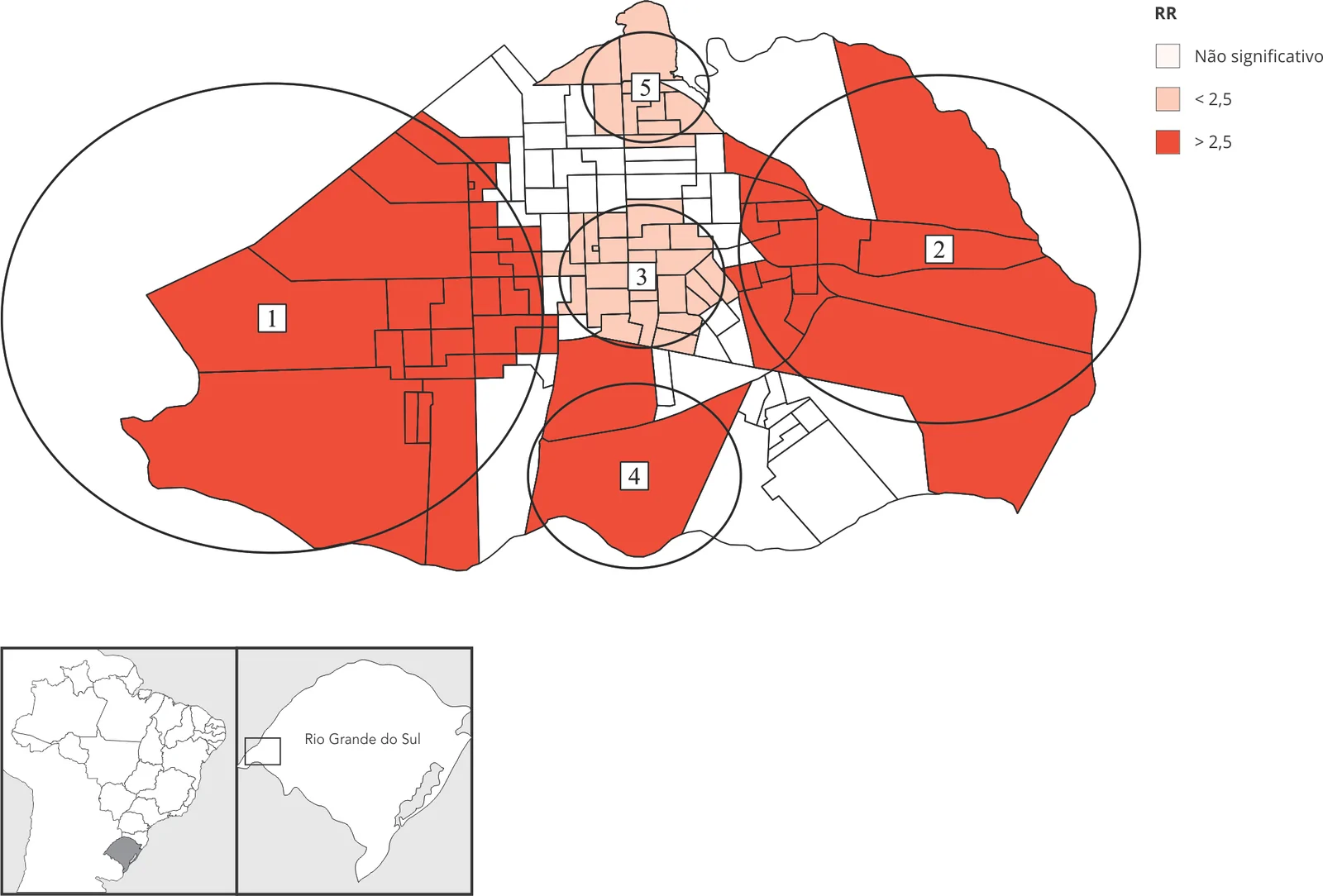
Aglomerados espaço-temporais de cães sororreagentes para leishmaniose visceral canina (LVC) identificados pelo modelo de varredura de Poisson no SaTScan. Município de Uruguaiana, Rio Grande do Sul, Brasil, 2009-2019.

DPP: domicílios particulares pemanentes; ELISA: *enzyme-linked immunosorbent assay*; RR: risco relativo; RIFI: reação de imunofluorescência indireta. Nota: foram considerados sororreagentes os cães positivos no protocolo diagnóstico vigente no período (DPP + RIFI entre 2009 e 2013; DPP + ELISA a partir de 2014). Foram destacados os aglomerados com RR superior a 2,5, ponto de corte adotado para identificar agrupamentos de maior relevância epidemiológica em análises exploratórias de vigilância espacial.

**Tabela 2:** Aglomerados espaço-temporais de cães sororreagentes para leishmaniose visceral canina (LVC) identificados pela estatística de varredura no SaTScan. Município de Uruguaiana, Rio Grande do Sul, Brasil, 2009-2019.

Aglomerado	Número de setores censitários	Número de DPP	Período	Casos observados	Casos esperados	RR	Valor de p (p < 0,05)
1	27	8281	2012-2015	297	133.57	2.55	< 0.001
2	15	4436	2012-2014	166	53.67	3.37	< 0.001
3	22	6323	2012-2014	147	76.51	2.02	< 0.001
4	2	619	2012-2016	41	12.48	3.35	< 0.001
5	9	2227	2011-2013	61	26.94	2.32	< 0.001

DPP: domicílios particulares permanentes; RR: risco relativo.

Nota: foram considerados sororreagentes os cães positivos no protocolo diagnóstico vigente em cada ano, composto pelo teste rápido DPP como triagem e ELISA (a partir de 2014) ou RIFI (entre 2009 e 2013) como teste confirmatório. A tabela apresenta o período de ocorrência de cada aglomerado, número de setores censitários envolvidos, número de DPP, período considerado, casos observados e esperados de cães sororreagentes, RR e valor de p obtido (p < 0,05).

## Discussão

### Situação epidemiológica de Uruguaiana no contexto nacional

Uruguaiana, embora classificada como área de transmissão estabelecida de LV ^20^, apresenta um cenário distinto de regiões endêmicas do Nordeste ^3^ ^,^ ^16^ e do Centro-oeste ^6^ ^,^ ^15^ ^,^ ^21^ ^,^ ^22^ brasileiros. A sororreatividade canina é elevada, mas a incidência humana permanece baixa e inferior à média nacional ^3^, caracterizando transmissão esporádica ^5^. No período de 2009-2019, três casos humanos foram confirmados frente a 1.478 cães sororreagentes, indicando predomínio da infecção no reservatório canino e sugerindo condições locais que podem não ter favorecido o transbordamento para humanos. Entre as hipóteses, destaca-se a possível predominância do comportamento zoofílico do vetor, o que reduziria o contato com seres humanos e contribuiria para a baixa transmissão.

### Papel do reservatório canino na dinâmica de transmissão da LV

A Figura 1 evidencia que a sororreatividade canina obtida nos inquéritos epidemiológicos realizados após casos humanos foi superior à encontrada nas coletas provenientes da demanda espontânea. Esse achado reforça o papel epidemiológico do cão como reservatório, uma vez que estudos prévios demonstram associação entre a ocorrência de LV em humanos e histórico de LVC no domicílio, constituindo fator de risco preditivo para a infecção humana ^6^ ^,^ ^13^ ^,^ ^23^ ^,^ ^24^ ^,^ ^25^. O controle do reservatório canino, portanto, é fundamental. Entre as medidas disponíveis, destaca-se o uso de coleiras impregnadas com deltametrina 4%, que reduzem a sororreatividade canina e a infecção dos flebotomíneos ^24^ ^,^ ^26^ ^,^ ^27^.

### Vigilância passiva, subnotificação e impacto nas análises espaço-temporais

As investigações provenientes de demanda espontânea (Tabela 1) mostraram aumento das coletas até 2013 e da sororreatividade até 2015, seguido por declínio no número de atendimentos. Essa redução coincide com a interrupção das ações de controle do reservatório canino pelo município a partir de 2014, o que pode ter diminuído a procura por diagnóstico no serviço público. Assim, a queda nas notificações reflete provavelmente subnotificação e não redução da circulação da infecção. Esse fenômeno também afetou a detecção de aglomerados, concentrados até 2016, limitando a avaliação espaço-temporal posterior. Municípios que interromperam ações continuadas de controle apresentaram tendências crescentes de incidência humana e sororreatividade canina ^6^ ^,^ ^8^ ^,^ ^24^, reforçando a importância da manutenção sistemática das atividades voltadas ao reservatório canino ^28^.

### Transformações ambientais, uso do solo e implicações no Bioma Pampa

As transformações ambientais do bioma Pampa podem influenciar a dinâmica de transmissão da LV, especialmente pela fragmentação da vegetação campestre e pela expansão agrícola. Entre 1985 e 2022, o Pampa Sul-americano perdeu cerca de 20% de sua cobertura de campo nativo, sendo o Brasil responsável pela maior redução proporcional ^29^. A alteração do uso do solo, associada ao avanço de culturas extensivas, tendem a modificar o hábitat de vetores e reservatórios silvestres, aumentando a interface entre fauna doméstica, vida silvestre e populações humanas. Esses processos podem favorecer ciclos zoonóticos e demandam monitoramento contínuo, sobretudo em municípios fronteiriços como Uruguaiana, onde mudanças ambientais podem repercutir no risco espacial da LV ^30^.

### Distribuição espaço-temporal e áreas prioritárias para intervenção

Durante o período do estudo, a LVC apresentou ampla distribuição pelos setores censitários de Uruguaiana, com identificação de cinco aglomerados espaço-temporais (Figura 2).

A presença desses aglomerados indica que a disseminação da LVC no município não ocorreu de forma homogênea, mas concentrada em determinados períodos e áreas do território urbano. A magnitude do risco relativo observada nessas áreas sugere intensificação local da transmissão em momentos específicos da série histórica. Esse padrão reforça a utilidade da estatística de varredura como ferramenta para identificar áreas prioritárias para vigilância e intervenção, especialmente em contextos de vigilância passiva, nos quais a distribuição dos registros pode refletir desigualdades no acesso ao diagnóstico.

Adicionalmente, a coincidência espacial entre alguns dos aglomerados identificados e a ocorrência de casos humanos reforçam a relevância epidemiológica da circulação prévia do parasito na população canina. Essa sobreposição evidencia a dependência espacial entre infecções em cães e humanos ^13^ ^,^ ^28^ e destaca áreas com maior risco potencial de transmissão ^7^, ressaltando a importância do monitoramento territorial da LVC como estratégia para antecipar situações de risco para a população humana.

### Limitações do estudo e desafios para a vigilância da LVC

O estudo apresenta limitações relacionadas à ausência de estimativas oficiais da população canina de Uruguaiana, o que impossibilita o cálculo de indicadores populacionais mais concretos e restringe a interpretação da sororreatividade observada. Os dados analisados referem-se majoritariamente à vigilância passiva, composta por cães cujos tutores buscaram o serviço público por apresentarem sinais clínicos compatíveis com LVC, o que pode ter contribuído para a elevada proporção de positividade registrada. Além disso, o banco de dados não incluiu atendimentos realizados em clínicas e hospitais veterinários privados, sugerindo que o número de casos no município possa ser maior do que o oficialmente notificado. Nesse contexto, iniciativas como o aplicativo desenvolvido por Massia et al. ^31^ representam estratégias promissoras para integrar registros públicos e privados, reduzir a subnotificação e aprimorar os sistemas de vigilância.

As taxas de cães por imóvel, frequentemente utilizadas como estimativa indireta da população canina na ausência de censos específicos, também apresentam limitações. Sua magnitude pode variar significativamente conforme o método de obtenção − como observação direta, cobertura vacinal, cadastros municipais ou amostragem por setor censitário − influenciando a interpretação do risco. No cenário de Uruguaiana, a falta de padronização dessas métricas reforça a necessidade de cautela nas comparações entre áreas e destaca a importância de aprimorar métodos de estimativa populacional que possam subsidiar ações de vigilância e controle mais precisas.

Adicionalmente, os dados provenientes dos inquéritos sorológicos não foram incluídos na análise espaço-temporal, uma vez que esses inquéritos apresentam cobertura territorial focalizada e não sistemática ao longo do período de estudo, o que poderia introduzir viés na identificação de aglomerados.

### Aspectos éticos, sofrimento moral e lacunas nas políticas públicas

Os desafios observados na implementação das ações de controle no município também dialogam com discussões mais amplas sobre as fragilidades estruturais das políticas públicas de enfrentamento da LVC no Brasil. A sobrecarga operacional e a limitação de ferramentas efetivas de manejo, especialmente em cenários de elevada sororreatividade canina e predominância da vigilância passiva, podem gerar sofrimento moral entre médicos veterinários − caracterizado pela impossibilidade de agir conforme os princípios éticos diante de barreiras institucionais ou legais. Conflitos recorrentes entre o bem-estar animal e as condições socioeconômicas dos tutores, como discutido por Mani ^32^, contribuem para o desgaste emocional desses profissionais. No contexto deste estudo, tais dilemas refletem as dificuldades de implementação contínua das ações de controle e evidenciam lacunas que comprometem a efetividade das políticas públicas voltadas ao enfrentamento da LVC.

### Saúde Única, Objetivos de Desenvolvimento Sustentável (ODS) e necessidade de políticas intersetoriais

As limitações observadas na vigilância da LVC em Uruguaiana reforçam a necessidade de políticas públicas mais integradas, alinhadas ao enfoque de Saúde Única. A elevada sororreatividade canina, associada às dificuldades de implementação de medidas como coleiras repelentes e ao manejo populacional, comprometem avanços em metas relacionadas à saúde e à vida terrestre (ODS 3 e ODS 15) ^33^. A integração entre saúde humana, animal e ambiental é essencial para orientar ações territorializadas e sustentáveis, especialmente em municípios fronteiriços com desigualdades estruturais. Nesse sentido, a articulação intersetorial e o fortalecimento da infraestrutura local podem contribuir para aprimorar o controle da LV e reduzir as vulnerabilidades evidenciadas ao longo do período estudado ^34^ ^,^ ^35^.

### Relevância do SIG para o planejamento territorial

Os resultados deste estudo reforçam o potencial das ferramentas de SIG para caracterizar a dinâmica espaço-temporal da LVC em Uruguaiana, permitindo identificar áreas de maior risco e a sobreposição entre casos caninos e humanos. No entanto, análises espaciais aplicadas à epidemiologia apresentam limitações inerentes associadas à qualidade dos dados, pressupostos estatísticos e variabilidade ambiental, aspectos que devem ser considerados na interpretação dos padrões observados ^36^. Essa cautela é especialmente relevante em contextos de vigilância passiva, como o deste estudo, nos quais a subnotificação pode influenciar a detecção de aglomerados e a compreensão da evolução temporal da infecção. Ainda assim, o emprego do SIG é estratégico para orientar ações territorializadas, apoiar o direcionamento de recursos e subsidiar políticas públicas mais ajustadas ao cenário epidemiológico local, sobretudo em municípios fronteiriços com desafios estruturais como Uruguaiana.

## Considerações finais

Os resultados deste estudo evidenciam que a LVC apresenta ampla distribuição territorial no Município de Uruguaiana, com ocorrência de aglomerados espaço-temporais que indicam a intensificação local da transmissão em determinados períodos da série histórica. A identificação desses padrões reforça o papel da vigilância epidemiológica centrada em análise espacial como ferramenta estratégica para reconhecer as áreas prioritárias para a intervenção e o controle.

A coincidência entre alguns dos aglomerados identificados e a ocorrência de casos humanos destaca a relevância do monitoramento da infecção canina como componente fundamental das estratégias de prevenção e controle da LVl. Nesse contexto, a utilização de ferramentas de geoprocessamento e análise espaço-temporal pode contribuir para o planejamento de ações territorializadas de vigilância, favorecendo a priorização de áreas de maior risco.

Os achados do estudo enfatizam a necessidade de fortalecer estratégias integradas de vigilância, capazes de articular diferentes fontes de informação e promover maior integração entre os serviços de saúde humana, saúde animal e vigilância ambiental. Iniciativas baseadas na abordagem Saúde Única podem contribuir para aprimorar o monitoramento da doença e orientar políticas públicas mais efetivas no enfrentamento da LV em contextos urbanos complexos.

## Data Availability

Os dados de pesquisa não estão disponíveis.

## Material Suplementar

Material Suplementar
