# Supplementary material for: A spatiotemporal analysis of canine visceral leishmaniasis in Uruguaiana, Rio Grande do Sul, Brazil (2009-2019)
Source: Cad Saude Publica. 2026 Oct 5;42:e00153625. [Article in Portuguese] doi: 10.1590/0102-311XPT153625 (PMC13640031; doi:10.1590/0102-311XPT153625)
Supplement: Supplementary file 1 [file 1678-4464-csp-42-PT153625-s.pdf]

Material Suplementar

Figura S1 Estimativa de densidade Kernel dos casos de leishmaniose visceral canina (LVC) no Município de Uruguaiana, Rio Grande do Sul, Brasil, 2009-2019.

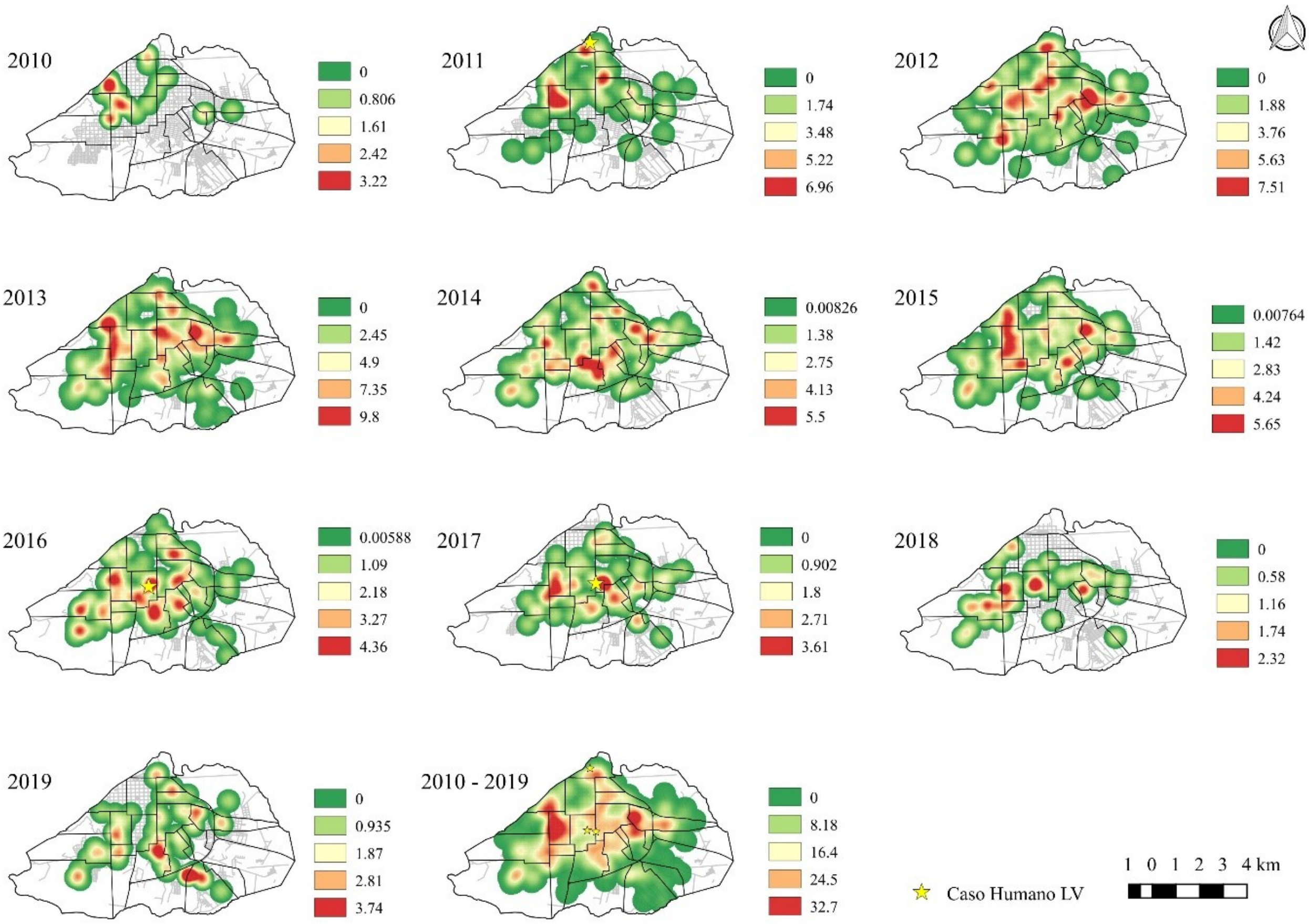

Figura S2 Análise de autocorrelação espacial local (Índice de Moran Local – LISA) dos casos de leishmaniose visceral canina (LVC) no Município de Uruguaiana, Rio Grande do Sul, Brasil, 2009-2019.

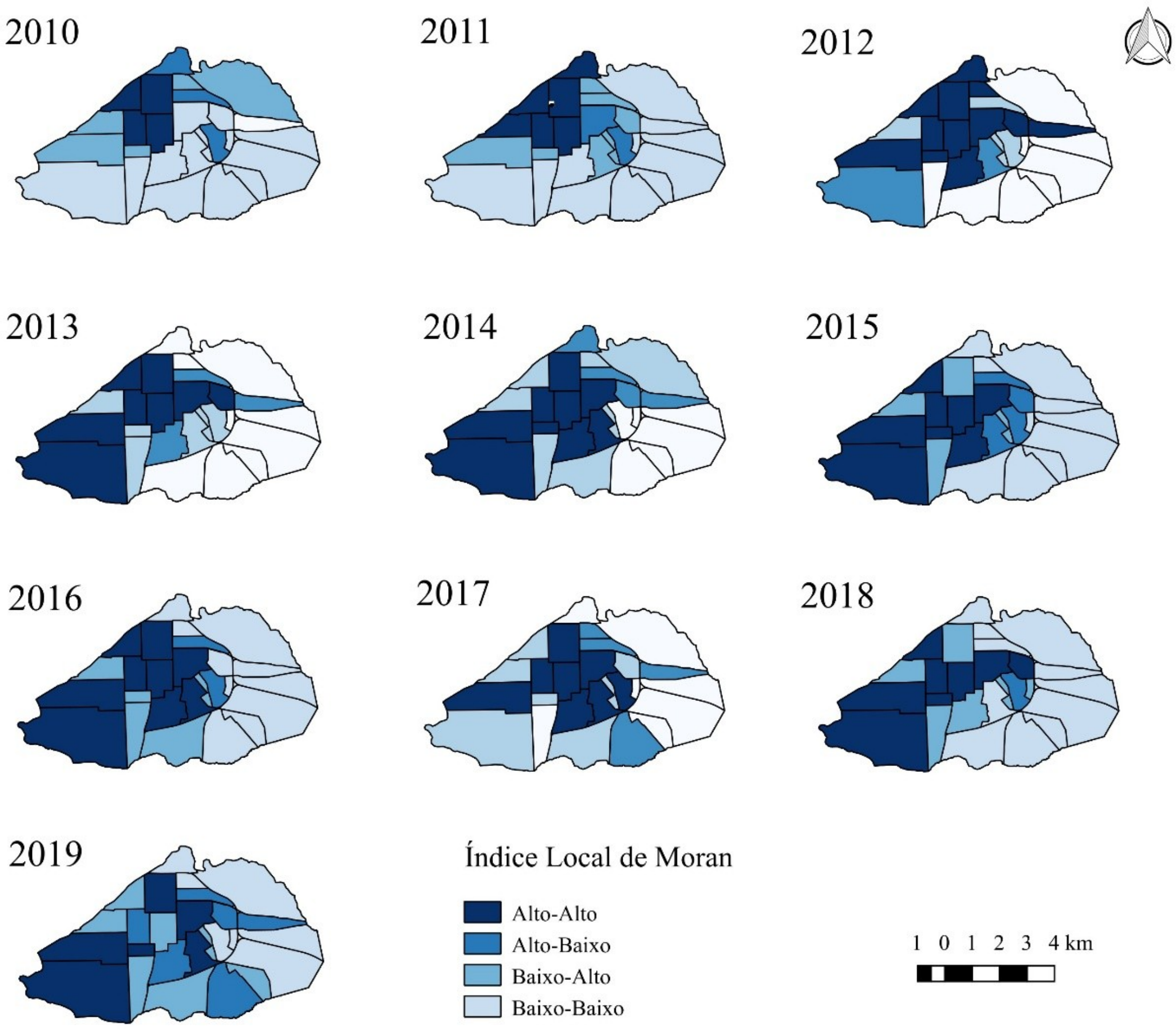

Quadro S1 Comparação entre os registros provenientes da demanda espontânea e do inquérito canino.

DIMENSÃO	DEMANDA ESPONTÂNEA	INQUÉRITO CANINO
**Tipo de vigilância**	Passiva	Ativa
**Fonte dos casos**	Procura espontânea dos tutores ao serviço municipal	Busca sistemática realizada pela equipe em setores definidos
**Viés**	Pode gerar viés de seleção devido à dependência da percepção do tutor	Menor viés; maior representatividade populacional
**Georreferenciamento**	Endereço informado pelo tutor	Endereço verificado pela equipe durante o inquérito
**Cobertura**	Irregular e dependente da demanda	Definida previamente por setor censitário
**Utilidade no estudo**	Complementar; indica procura por sinais clínicos da doença	Base principal da análise espacial e espaço-temporal
